# The Reflexes in Recent Hemiplegia

**Published:** 1909-02-13

**Authors:** 


### THE REFLEXES IN RECENT HEMIPLEGIA.

It is well enough known that in a recent case of hemiplegia due to some such cause as cerebral hæmorrhage the knee-jerk upon the paretic side may be absent, or less brisk than, equal to, or greater than that upon the sound side. Further, it is possible to have at the same time increased knee-jerk and flexor plantar reflex; or increased knee-jerk and extensor plantar reflex (Babinski's sign); or diminished or absent knee-jerk with extensor plantar reflex. These variations in the reflexes in recent hemiplegia have been much studied, and certain general inferences can probably be drawn from them. Touche concludes as follows:

The commencement of the hemiplegia is marked by an exaggeration in the knee-jerk upon the affected side, extending in some instances to the sound side also. The plantar reflex is equally exaggerated in the earlier stages of the hemiplegia, and it is accompanied by extension of the big toe on the paralysed side. After a certain time, varying from a few hours to several days, the exaggeration of the knee-jerk is prone to change to abolition. The same phenomenon occurs also in the case of the plantar reflex, but the disappearance of the extensor plantar reflex is delayed, often until several days after the knee-jerk has disappeared. Death is very liable to occur before this Babinski's sign has entirely gone. The co-existence in a case of recent hemiplegia of absence of knee-jerk with presence of Babinski's sign is an association of very grave prognostic omen. As a rule there is also complete coma and marked elevation in temperature.

The early abolition of the knee-jerk without co-existent Babinski's sign, together with the preservation of a good general condition without pyrexia, would indicate a transitory and entirely curable hemiplegia, such as is not very uncommon in elderly gentlemen with arterio-sclerosis. When hemiplegia attacks a patient whose reflexes are already exaggerated owing to pre-existent disease of the brain, medulla, or cord, that exaggeration persists or even becomes increased in the earlier phases of the hemiplegia, to diminish later and finally disappear just as, under similar circumstances, in subjects whose reflexes have hitherto been quite normal.

If the reflexes were absent before the stroke, the hemiplegia may cause them to reappear for a time, though presently they will go again exactly as they would in normal subjects.

Post-mortem examination suggests that the exaggeration of reflexes of the early stage of hemiplegia is related to a moderate hæmorrhage exercising simply an irritant action upon the pyramidal fibres, whilst their final abolition, accompanied by coma and pyrexia, indicates their total and acute destruction.

